# Colorectal cancer patients with *CASK* promotor heterogeneous and homogeneous methylation display different prognosis

**DOI:** 10.18632/aging.103928

**Published:** 2020-10-28

**Authors:** Ying Liu, Hao Huang, Jinming Fu, Yuanyuan Zhang, Jing Xu, Lei Zhang, Simin Sun, Liyuan Zhao, Ding Zhang, Justina Ucheojor Onwuka, Hongru Sun, Binbin Cui, Yashuang Zhao

**Affiliations:** 1 Department of Epidemiology, Public Health College, Harbin Medical University, Nangang District, Harbin 150086, Heilongjiang Province, The People’s Republic of China; 2 Department of Colorectal Surgery, The Affiliated Tumor Hospital of Harbin Medical University, Harbin 150086, Heilongjiang Province, The People’s Republic of China

**Keywords:** heterogeneous methylation, *CASK*, colorectal cancer, prognosis, dMS-HRM

## Abstract

Homogenous DNA methylation clearly affects clinical outcomes. However, less is known about the effects of heterogeneous methylation. We aimed to investigate the different effects between *CASK* promoter methylation heterogeneity and homogeneity on colorectal cancer (CRC) patients' prognosis. The methylation status of *CASK* in 296 tumor tissues and 255 adjacent normal tissues were evaluated using Methylation-sensitive high-resolution melting (MS-HRM). Digital MS-HRM (dMS-HRM) visualized heterogeneous methylation and subsequent sequencing provided exact patterns. Log-rank test and Cox regression model were adopted to assess the association between *CASK* methylation status and CRC prognosis with propensity score (PS) method to control confounding biases. Heterogeneous methylation was detected in both tumor (52.2%) and non-neoplastic tissue surrounding the tumor (62.4%). It occurred more frequently in lower levels of tumor invasion (*P* = 0.002) and male patients (*P* < 0.001). Compared with heterogeneous methylation, patients with *CASK* homogeneous methylation presented poorer overall survival (OS) (HR: 1.919, 95% CI: 1.146-3.212, *P* = 0.013) and disease-free survival (DFS) (HR: 1.913, 95% CI: 1.146-3.194, *P* = 0.013). This unfavorable effect still existed among older (≥ 50), Dukes staging C/D, and rectal cancer patients. MS-HRM and dMS-HRM when combined can assess the degree and complexity of heterogeneous methylation with a visible pattern.

## INTRODUCTION

Colorectal cancer (CRC) is a commonly diagnosed malignant neoplasm which ranks third in terms of incidence and second in terms of mortality in the world [1]. Currently, the main treatment for patients with CRC remains surgical resection [2], postoperative radiotherapy [3], and chemotherapy [4]. In developed countries, overall CRC survival rates are improving because of major improvements in early diagnosis and therapy. The 5-year relative survival rate for CRC was 65% (colon was 64%, rectum was 67%) in the United States in 2019 [5], while in developing countries it is still poor for most CRC patients [6]. Three systems are used for the staging of CRC: Astle-Coller system, TNM system and Dukes staging [7]. Among them, Dukes staging is currently recognized as the most reliable indicator for assessing CRC patient’s prognosis [8]. However, the Dukes staging is not sensitive and accurate enough to evaluate CRC prognosis and recurrence [9]. Thus, it is necessary to investigate new predictive biomarkers.

Several studies have provided evidence that aberrant DNA methylation is associated with CRC outcomes [10–13]. Aberrant DNA methylation of CpG islands correlates with epigenetic gene silencing and drives tumor progression through key signaling pathways [14]. Consequently, aberrant DNA methylation has the potential to be a prognostic and predictive biomarker for CRC [8]. However, studies on promoter aberrant DNA methylation mainly focused on the detection of methylation degree while heterogeneous DNA methylation has rarely been further investigated.

Unlike homogeneous methylation, heterogeneous DNA methylation is referred to as multiple alleles with different DNA methylation patterns [15], each with a varied number of CpG sites methylated. It is the intermediate stage between fully methylated and unmethylated [16]. More so, because the methylation of individual CpG sites is not faithfully maintained by DNA methyltransferase [17], the methylation of alleles is incomplete and could reflect epigenetic instability [18]. To date, studies on the relationship between heterogeneous methylation and tumorigenesis [17–21] demonstrated that heterogeneous methylation might appear early in the progression of the tumor [19] and correlate with gene silencing. It has been proved that age [22], environmental factors [23–25], cell division [26], and other cancer-associated phenomena [27, 28] are contributing factors in the development of heterogeneous methylation.

*CASK* encodes the calcium/calmodulin-dependent serine protein kinase which is a member of the MAGUK (membrane-associated guanylate kinase) protein family. As the scaffold protein, it can couple diverse signal transduction pathways [29]. *CASK* is able to bind with four cancer-related cell adhesion receptors (syndecan-1, -2, -3, and -4) to adjust cell proliferation, migration, invasion, and gene expression through the signal pathway [29–34]. It is worth noting that *CASK* is associated with tumor activity inhibition as the crucial regulator in the carcinogenesis of CRC [30, 34, 35]. *CASK* has been reported to be over-expressed in CRC [36], gastric cancer [37], and esophageal carcinoma [38]. Moreover, there is an association between high expression of *CASK* and unfavorable prognosis of colorectal cancer [36]. However, there is no evidence to illustrate the relationship between *CASK* methylation patterns and the prognosis of patients with CRC, especially the difference in prognosis between heterogeneous and homogeneous methylation patients.

Here, we comprehensively investigated the different effects of heterogeneous and homogeneous methylation of *CASK* on CRC prognosis in a long-term cohort using the propensity score (PS) adjusted method [39] to control the confounders. In addition, dMS-HRM was used for the visualization and interpretation of complex heterogeneous methylation patterns [40].

## RESULTS

### MS-HRM analyses of samples

**Figure 1:**
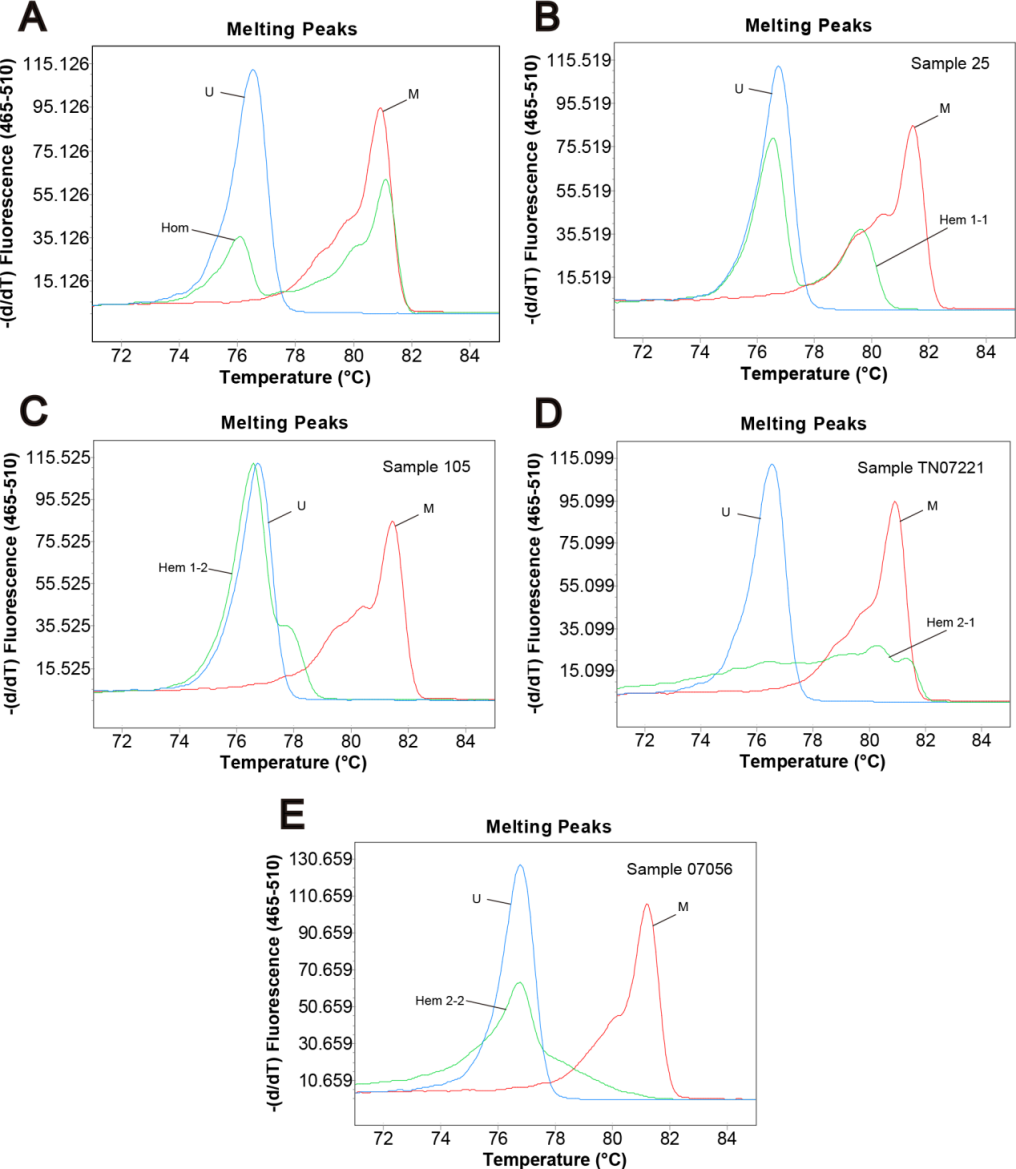
**Methylation status of CRC samples defined by melting peak.** Melting profiles of the methylation control (M), unmethylation control (U), and (**A**) the sample with homogeneous methylation (Hom); (**B**) sample 25 with heterogeneous methylation 1-1 (Hem 1-1); (**C**) sample 105 with heterogeneous methylation 1-2 (Hem 1-2); (**D**) sample TN07221 with heterogeneous methylation 2-1 (Hem 2-1); (**E**) sample 07056 with heterogeneous methylation 2-2 (Hem 2-2).

Genomic DNA from 296 primary tumor tissues and 255 adjacent normal tissues of CRC patients were analyzed for methylation status by MS-HRM. Among them, 107 (47.8%) tumor samples and 65 (37.6%) adjacent normal tissue samples were defined as homogeneous methylation. In addition, we also found heterogeneously methylated samples. Their melting profiles do not conform to homogeneous methylation. Figure 1A–1E shows the typical MS-HRM melting curves of *CASK* heterogeneous methylation. The characteristic melting pattern of heterogeneous methylation indicated that the products derived from heterogeneously methylated samples were not a homogeneous mixture of fully methylated and fully unmethylated sequences.

### Post MS-HRM analyses

**Figure 2:**
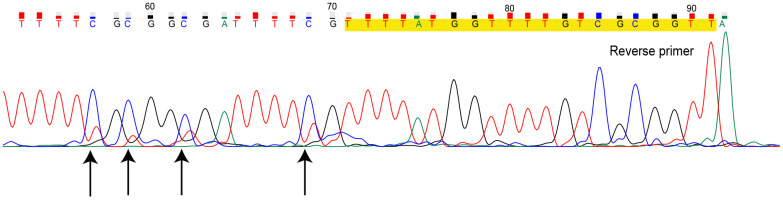
**Direct sequencing for methylation-sensitive high-resolution melting (MS-HRM) product of sample TN07221.** Primers are marked in yellow, black arrows indicate CpG sites with overlapping peak. (Dye blobs in the unidirectional Sanger sequencing result in uncertainty of bases at the beginning of sequence giving incomplete coverage. Hence, we only provided the definite part of the entire sequencing result.)

The PCR products of MS-HRM for heterogeneously methylated samples can be further investigated. Direct sequencing of the MS-HRM product for TN07221 (Hem 2-1) is illustrated in Figure 2. Direct sequencing presented the overall readout across the entire amplicons. The overlapping peaks indicated that the sites were a mixture of cytosine and thymine.

**Figure 3:**
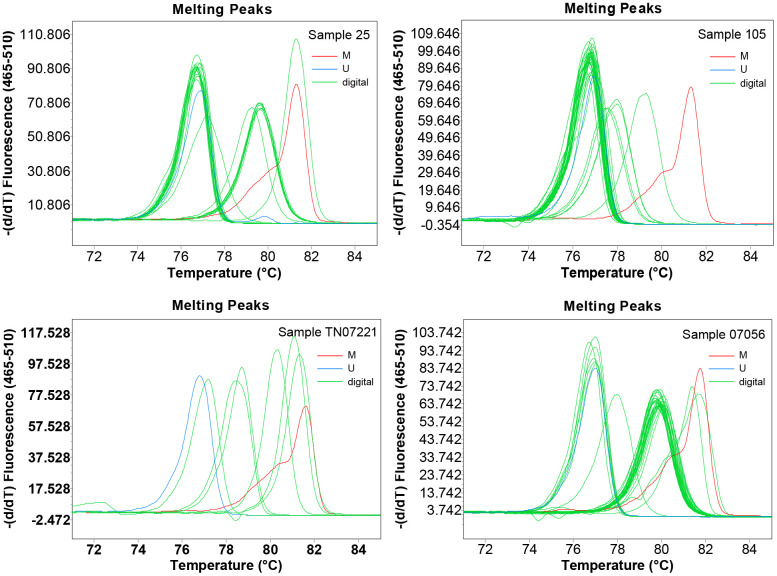
**The visualized methylation profiles of four samples performed using digital methylation-sensitive high-resolution melting (dMS-HRM).** M: methylation control; U: unmethylation control; digital: digital output of amplicons.

**Figure 4:**
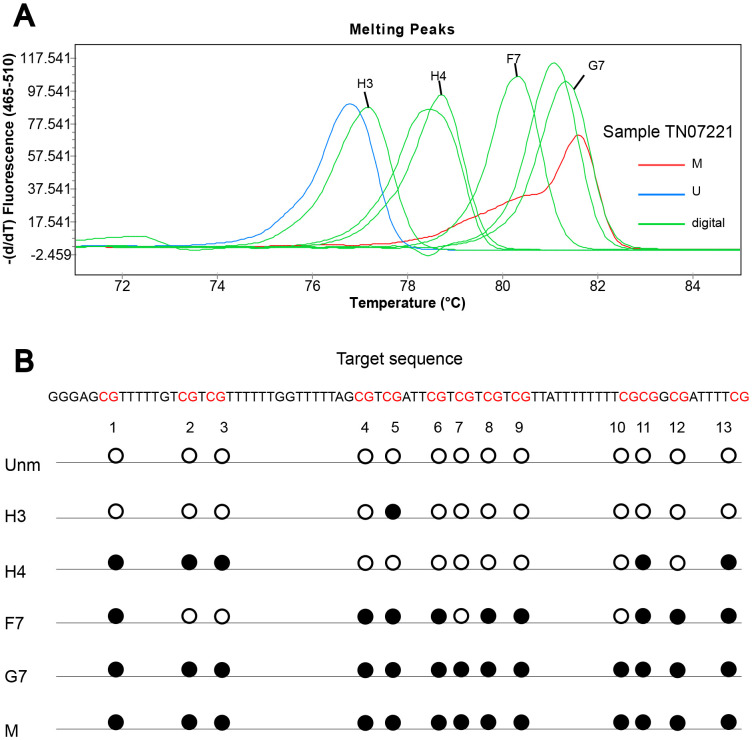
**Sanger sequencing of digitally obtained clones from sample TN07221.** (**A**) Products numbered H3, H4, F7 and G7 were sequenced; (**B**) solid circle and hollow circle illustrate methylated and unmethylated CpG sites, respectively.

To obtain detailed profiles of methylation pattern, dMS-HRM was performed to exactly visualize the individual epiallele. Sample 25 (Hem 1-1), 105 (Hem 1-2), TN07221 (Hem 2-1), and 07056 (Hem 2-2) (Figure 1), representing four kinds of heterogeneous methylation, were analyzed digitally. Figure 3 displays the visualized methylation profiles of the selected samples. They all contained multiple heterogeneously methylated alleles and melted unevenly. We chose dMS-HRM products of sample TN07221 with Hem 2-1 heterogeneous methylation for subsequent Sanger sequencing because of its wide range of variation. It was also the best sample to illustrate the correlation between peak position and the degree of methylation. As the quantities of methylated CpG sites in allele increased, the Tm also increased, and the position of the peak was closer to methylated control. Sanger sequencing of digitally obtained clones is shown in Figure 4 as the validation.

### Different distribution of heterogeneous and homogeneous methylation

We found that heterogeneous methylation was not only in the tumor, but also in non-neoplastic tissue surrounding the tumor. The proportion of heterogeneous methylation in adjacent normal tissue (62.4%) was higher than the tumor (52.2%) (Supplementary Table 1). Furthermore, heterogeneous methylation of *CASK* occurred more frequently in CRC patients with lower levels of tumor invasion (T1-T3, *P* = 0.002) and male patients (*P* < 0.001) (Supplementary Table 2). And the different distribution of heterogeneous and homogeneous methylation between tumor tissues and adjacent normal tissue samples was statistically significant (*P* = 0.042) (Supplementary Table 1).

**Table 1:** Characteristics of study subjects and comparisons of survival time between groups.

**Baseline characteristics**	**Overall survival time (months)**		***P*^‡^**	**Disease-free Survival (months)**		***P*^‡^**
	**Mean (SE)**	**Median^†^**		**Mean (SE)**	**Median^†^**	
Age						
< 50	71.457 (5.077)	83		66.331 (5.613)	58	
≥ 50	78.786 (2.545)	> 102	0.220	76.296 (2.744)	> 102	0.135
Gender						
Male	78.680 (2.933)	> 83		75.413 (3.210)	> 96	
Female	74.833 (3.620)	96	0.470	72.496 (1.476)	96	0.478
Primary site						
Colon	78.532 (3.907)	> 98		76.149 (4.165)	> 98	
Rectum	76.547 (2.856)	96	0.482	73.314 (3.129)	102	0.522
Dukes staging						
A/B	89.056 (2.574)	>102		86.734 (2.889)	>102	
C/D	63.337 (3.615)	58	**< 0.001**	59.721 (3.819)	47	**< 0.001**
TNM staging						
I/II	89.126 (2.624)	> 96		87.299 (2.886)	> 96	
III/IV	62.504 (3.553)	57	**< 0.001**	58.752 (3.764)	47	**< 0.001**
Tumor invasion						
T1-T3	83.660 (2.995)	> 98		80.996 (3.303)	> 98	
T4	72.234 (3.490)	81	**0.013**	68.969 (3.731)	81	**0.021**
Lymph node metastasis						
N0	88.315 (2.659)	> 96		86.520 (2.907)	> 96	
N1/N2	62.911 (3.580)	58	**< 0.001**	59.061 (3.802)	47	**< 0.001**
Distant metastasis						
M0	79.819 (2.275)	> 102		77.058 (2.482)	> 102	
M1	25.308 (6.284)	18	**< 0.001**	24.615 (6.413)	18	**< 0.001**
Histological grade^§^						
G1/G2	80.841 (2.413)	> 102		78.146 (2.640)	> 102	
G3/G4	61.493 (6.719)	63	**0.006**	57.659 (7.107)	43	**0.003**
Histological type^§^						
Adenocarcinoma	76.451 (2.597)	98		73.266 (2.827)	102	
Other types	80.805 (4.687)	> 75	0.559	78.366 (5.069)	> 69	0.476
Pathological classification^§^						
Protuberant	84.870 (2.600)	> 94		82.224 (2.857)	> 94	
Other types	66.478 (4.222)	71	**< 0.001**	63.034 (4.523)	63	**< 0.001**
Preoperative CEA						
0-5ng/ml	86.999 (3.161)	> 75		86.425 (3.256)	> 96	
≥ 5ng/ml	70.345 (3.092)	80	**< 0.001**	65.720 (3.396)	71	**< 0.001**
Preoperative CA19-9						
0-37U/ml	90.190 (2.293)	> 102		88.674 (2.468)	> 102	
≥ 37U/ml	44.764 (3.591)	36	**< 0.001**	37.710 (3.812)	26	**< 0.001**
Postoperative chemotherapy						
No	77.694 (3.136)	102		77.258 (3.172)	> 102	
Yes	77.231 (3.329)	> 96	0.988	70.847 (3.902)	> 96	0.414
Postoperative radiotherapy						
No	78.283 (2.481)	> 96		75.990 (2.652)	> 102	
Yes	49.337 (8.017)	46	**0.004**	38.198 (9.017)	25	**0.001**
Postoperative biotherapy						
No	76.212 (2.591)	102		73.685 (2.770)	> 102	
Yes	88.329 (4.772)	> 80	0.075	83.256 (5.866)	> 69	0.143
Methylation status						
Unm	77.940 (4.469)	> 83		74.401 (4.953)	> 81	
Tpm	76.956 (2.635)	102	0.802	74.202 (2.842)	> 102	0.834
Hom	70.179 (3.904)	80		68.150 (4.158)	94	
Thm	82.172 (3.511)	> 98	**0.023**	79.248 (3.825)	> 98	**0.030**

^†^The mean survival time was underestimated because the largest observation was censored and the estimation was restricted to the largest event time.

^‡^*P* value was calculated using Log-tank test for comparing survival rates between two groups.

^§^G1-highly differentiated, G2-moderately differentiated, G3-poorly differentiated, G4-undifferentiated. Other types of histological type include mucinous adenocarcinoma, undifferentiated carcinoma, squamous cell carcinoma, papillary adenocarcinoma, signet ring cell carcinoma and other types. Other types of pathological classification include ulcer, invasion, narrow, nodular and other types.

### Association between different *CASK* methylation status and prognosis of CRC

The mean and median survival times for all subjects are shown in Table 1. Patients with homogeneous methylation presented statistically significant lower survival rates. The 8-year OS rate was 27% in homogeneous methylation group versus 56% in heterogeneous methylation group (*P* = 0.023) (Supplementary Table 3). When compared with unmethylation, homogeneous methylation predicted more unfavorable survival (HR_multivariate-adjusted_: 2.473, 95% CI: 1.136-5.382, *P* = 0.022); however, there was no statistically significant association between heterogeneous methylation (Tpm and Thm) and CRC prognosis (Supplementary Table 4).

We found that the effects of homogeneous and heterogeneous methylation on CRC prognosis were distinctly different. The results of univariate and multivariate Cox analysis demonstrate that patients with *CASK* homogeneous methylation presented poorer OS and DFS compared with heterogeneous methylation (Supplementary Tables 5, 6). The Kaplan-Meier survival curves are shown in Supplementary Figure 1. In addition, compared with Hem 1-1 and Hem 2-1 respectively, the unfavorable effect of *CASK* homogeneous methylation on CRC prognosis also persisted (Supplementary Table 6).

We further performed PS regression analysis to minimize the potential effects of confounding factors [16 factors including age, gender, primary site, staging, etc. (details are shown in baseline characteristics of Table 1)], and to assess the effect estimates between categorical variables and the outcome variables [41]. Compared with heterogeneous methylation, the association between *CASK* homogeneous methylation and unfavorable survival was still statistically significant. The HR_PS-adjusted_ for OS was 1.919 (95% CI: 1.146-3.212, *P* = 0.013) and for DFS was 1.913 (95% CI: 1.146-3.194, *P* = 0.013) (Table 2).

**Table 2:** Comparison of the unadjusted and the adjusted effect estimates (Hom vs Thm).

**HR_s_ for *CASK* methylation and CRC prognosis**	**HR (95% CI)**	***P*^†^**	**confounding RR^‡^**	***P*^§^**
OS				
Unadjusted	1.603 (1.062-2.419)	**0.025**		
Variable adjusted	2.501 (1.383-4.525)	**0.002**	1.560	0.227
PS-adjusted	1.919 (1.146-3.212)	**0.013**	1.197	0.594
DFS				
Unadjusted	1.567 (1.039-2.364)	**0.032**		
Variable adjusted	2.495 (1.394-4.464)	**0.002**	1.592	0.201
PS-adjusted	1.913 (1.146-3.194)	**0.013**	1.221	0.552

^†^*P* value was calculated using Cox regression analysis.

^‡^Confounding RR was defined as the ratio of the PS-adjusted HR and the unadjusted HR or the variable-adjusted HR and the unadjusted HR.

^§^Test for heterogeneity between HRs was conducted by Comprehensive Meta-Analysis (version 2.0).

### Subgroup and sensitivity analysis

According to the result of the subgroup analysis (Table 3), we observed that the disadvantageous effect of *CASK* homogeneous methylation on survival was still significant among older (≥ 50), Dukes staging C/D, and rectal cancer patients. Next, we compared unadjusted and adjusted effect estimates using "confounding RR", and found there was no heterogeneity between them (*P* > 0.050) (Table 2).

**Table 3:** Subgroup analysis for association between *CASK* methylation status and CRC prognosis (Hom vs Thm).

**Subgroup**	**Hom, No**	**Thm, No**	**PS-adjusted^†^ (OS) HR (95% CI)**	***P*^‡^**	**PS-adjusted^†^ (DFS) HR (95% CI)**	***P*^‡^**
Gender						
Male	20	83	1.754 (0.826-3.724)	0.144	1.770 (0.838-3.738)	0.135
Female	87	34	1.928 (0.949-3.917)	0.070	1.915 (0.949-3.868)	0.070
Age						
< 50	20	26	1.147 (0.383-3.435)	0.806	1.216 (0.405-3.654)	0.728
≥ 50	87	91	2.299 (1.277-4.141)	**0.006**	2.143 (1.196-3.838)	**0.010**
Tumor site						
Rectal	65	72	2.012 (1.050-3.855)	**0.035**	1.972 (1.034-3.763)	**0.039**
Colon	42	45	1.780 (0.762-4.156)	0.183	1.805 (0.777-4.195)	0.170
Dukes staging						
A/B	64	59	1.688 (0.682-4.080)	0.262	1.663 (0.680-4.071)	0.265
C/D	43	58	2.138 (1.153-3.964)	**0.016**	2.196 (1.196-4.032)	**0.011**

^†^HR values were the effect estimates adjusted by propensity score.

^‡^*P* value was calculated using Cox regression model.

### Association between different *CASK* methylation status and prognosis of CRC in the validation TCGA dataset

We evaluated the methylation status of the three probes (cg02161125, cg03983969, and cg12614178) annotated by HM450 and located in the target sequence of our study in 379 CRC tissues. The methylation data were downloaded from TCGA Data Portal (http://tcga-data.nci.nih.gov/tcga/) [42]. We defined samples with high different methylation levels (cutoff was set according to the SD for beta values of three probes) of the three adjacent sites as heterogeneous methylation, otherwise as homogenous methylation. At last, 169 samples were defined as heterogeneous methylation (169/379). We also observed a tendency that homogeneous methylation had poorer survival than heterogeneous methylation even without statistical significance (HR_multivariate-adjusted_: 1.700, 95% CI: 0.819-3.532, *P* = 0.155) (Supplementary Table 7). Subgroup analysis also had the same trend as our initial findings (Supplementary Table 8).

## DISCUSSION

To the best of our knowledge, this is the first study to reveal the different effects between *CASK* promoter heterogeneous and homogeneous methylation on the prognosis of CRC patients. MS-HRM allowed us to evaluate heterogeneous methylation in a large sample and minimize analysis costs. The dMS-HRM performed a detailed analysis of representative samples. It also enabled us to obtain detailed heterogeneous methylation patterns both cost and time-effectively [40]. The PS-adjusted method can substantially minimize the number of independent variables included in the regression model and reduce the bias caused by collinearity [43].

Our results indicated that heterogeneous methylation of *CASK* promoter was distinctly different from homogeneous methylation on CRC prognosis, and homogeneous methylation patients with a more unfavorable prognosis. Furthermore, the results of multivariate Cox analysis and PS regression adjusted analysis suggested that this association was not affected by known confounding factors. The results were also verified to some extent by data from the TCGA database. Additionally, the unfavorable association was also observed in the older (≥ 50), Dukes staging C/D, and rectal cancer patients. There was no statistically significant association in the subgroup analysis based on gender, which may be due to our limited sample size. We confirmed there was no evidence of heterogeneity by assessing confounding RR. Based on the above analyses, our results were reliable to identify the different effects of *CASK* promoter heterogeneous methylation and homogeneous methylation on prognosis. We also found a weak correlation (r = -0.106, *P* = 0.038) between the methylation level of *CASK* (cg12614178) and the expression level in the TCGA database (Supplementary Figure 2). And, it has been proven that high expression of *CASK* is associated with poor prognosis of CRC [36]. It is reasonable that we speculate *CASK* methylation may affect the prognosis of CRC via gene expression. Further investigation is needed.

In the current study, we found that both tumor and non-neoplastic tissue surrounding the tumor presented heterogeneous methylation. This validated the findings of Azhikina et al. in previous study [19]. The heterogeneity of methylation appeared more in normal tissue surrounding tumor. Methylation of cancer-related genes in promoter regions may initially occur in normal tissues adjacent tumors. It may imply an early stage before pathological changes. More so, heterogeneous methylation is considered as the intermediate stage between fully methylation and unmethylation [16]. As was reported by previous research, during cancer progression from a benign tumor to malignancy, a single CpG site is independently methylated, and the methylation of specific CpG sites is "seeded" over time until all CpG sites are methylated [44]. Thus, it is reasonable that we found heterogeneous methylation was more likely to occur in patients with lower levels of tumor invasion (T1-T3).

When DNA methylation is regarded as a cancer biomarker, methylation heterogeneity is generally not taken into consideration. The methylation level is mostly reported as an average of the entire amplicon. Moreover, heterogeneous methylated samples are often ignored or sometimes mistaken for homogeneous methylation or unmethylation. Consequently, the correct interpretation of the methylation patterns will be compromised, and some reports may have misestimated the frequency of methylation events. Studies about the effects of positive methylation should pay attention to different methylation types and the difference between them to get a correct and comprehensive interpretation.

Previous studies found that many promoter regions of genes were heterogeneously methylated, such as *GHSR* in breast cancer [20], *CDKN2B* in acute myeloid leukemia [40], *DAPK1* and *LPL* in chronic lymphocytic leukemia [45, 46], *RARB* [47] and *SOX18* [19] in non-small cell lung cancer, and *ABO*, *RUNX3*, *CDH1*, *CDH13* in oral tongue squamous cell carcinomas [18]. Nevertheless, the evaluation of heterogeneous DNA methylation has not been performed extensively in a relatively large and long-term cohort. Also, few investigations have been focused on the clinical consequences of this process. The study on chronic lymphocytic leukemia evaluated the relationship between heterogeneous methylation and time to treatment (TTT) [46]. It indicated that patients with *LPL* full (8/112) or heterogeneous methylation (64/112) predicted longer TTT. The study on oral tongue squamous cell carcinomas assessed the effect of heterogeneous methylation on survival which found that patients with *RUNX3* heterogeneous methylation (18/108) had a worse survival than unmethylation [18]. Consequently, the effect of heterogeneous methylation on prognosis may vary due to different gene and cancer.

Many techniques are not suitable to evaluate heterogeneous methylation. Direct sequencing and pyrosequencing can estimate the average methylation degree at each CpG site, while the number of methylated templates cannot be inferred. Thus, the explanation of the results is often challenging [48]. Bisulfite sequencing of the individual clone is effective but expensive and strenuous [17, 49]. Massively parallel deep sequencing is an exact research technique, but it is high-cost and complex [50, 51]. Denaturing Gradient Gel Electrophoresis can visualize heterogeneous methylation [52, 53], droplet digital PCR (ddPCR) can be used to identify and quantify heterogeneously methylated epialleles as a new technique [21], and EpiHRMAssay can provide additional information for assessing heterogeneous methylation [54]. But these techniques are expensive and have not been widely used.

MS-HRM assays are able to identify heterogeneously methylated samples readily [55]. As a fast, sensitive, reliable, and robust method for distinguishing heterogeneous methylation, MS-HRM can qualitatively estimate heterogeneous methylation. Digital PCR of a single template eliminates cloning bias and PCR bias well [56]. Consequently, dMS-HRM is an effective tool to precisely identify methylation patterns which will be confirmed by subsequent sequencing. We combined these two methods to assess the *CASK* heterogeneous methylation. The representative samples pre-screened by MS-HRM were performed for dMS-HRM. The comprehensive methylation pictures of the target sequence were provided. Aimed to provide an interpretable visualization of heterogeneous methylation and reveal the heterogeneous methylation status of each individual CpG dinucleotide, we performed sequencing. Our findings showed that the products of heterogeneously methylated amplicons were composed of alleles that only differ in a few CpG sites, and Tm increased with quantities of methylated CpG sites in allele. In keeping with our result, previous studies reported that heterogeneously methylated samples led to extensive heteroduplex formation. The formation of heteroduplexes was due to the presence of molecules that differ by only a few bases [40] [45].

Our research also has several limitations. Above all, heterogeneous methylation can only be estimated in a qualitative manner. However, MS-HRM is a specific, sensitive, and economical method that is most suitable for detecting heterogeneous methylation. And heterogeneous methylation patterns were further assessed by subsequent analyses (dMS-HRM and sequencing) in this research.

Furthermore, it is not clear that the mechanisms of which *CASK* promoter heterogeneous and homogenous methylation differently affect the prognosis of CRC. To our knowledge, we show for the first time the different effects of homogeneous and heterogeneous methylation on the prognosis of CRC patients. A hypothesis has been proposed that heterogeneous methylation may not be sufficient to eliminate the transcription like homogeneous methylation, but may only interfere with the transcription process or be a "passenger" of the carcinogenesis [20]. More so it has been proven that various levels of methylation density of CpG sites correlated with the status of gene silencing [17]. The mechanism for the different prognostic effects of heterogeneous and homogeneous methylation is still unclear and further studies are still essential.

Currently, digital PCR assays are frequently used in liquid biopsies to investigate DNA methylation biomarkers [57–59]. But it cannot accurately quantify heterogeneous DNA methylation. Particularly, all methylated clones need to be sequenced when further investigate heterogeneous methylation. It high-cost and difficult to analyze heterogeneous methylation comprehensively [15]. Thus, the role of heterogeneous methylation in cancer diagnosis and prognosis prediction is usually neglected. Meanwhile, dMS-HRM can easily identify and screen single template clones by melting curves. It can significantly reduce the amount of sequencing and obtain exhaustive methylation patterns. Therefore, dMS-HRM which uses low copy and heterogenous methylated DNA in liquid biopsies might be expected to be applied in CRC diagnosis, prognosis evaluation, prediction of therapeutic efficacy, and recurrence monitoring. The future development of biomarkers that combined homogeneous methylation status and heterogeneity will improve comprehensive outcome prediction.

Future research should pay more attention to the presence of heterogeneous methylation when analyzing the methylation level of the specific gene sequences. It is inadvisable to ignore heterogeneous methylation or treat them as homogenous methylation when analyzing prognosis.

## CONCLUSIONS

CRC patients with homogenous methylation in *CASK* promoter showed a more unfavorable prognosis than those with heterogeneous methylation. Combine MS-HRM and dMS-HRM can identify and visualize heterogeneous methylation patterns.

## MATERIALS AND METHODS

### Patients and tissue specimens

This study included primary CRC patients who were diagnosed at the Third Affiliated Hospital of Harbin Medical University between November 2004 and January 2008. All tumor specimens were obtained after participants provided informed consent. Tumors and adjacent normal tissues were excised at the time of surgery and rapidly frozen in liquid nitrogen. They were recognized by two independent senior pathologists and then stored at -80°C refrigerator in the laboratory. We also collected clinicopathological data based medical records (Table 1). The study is consistent with the Declaration of Helsinki in 2000. All procedures performed in this study are in accordance with the ethical standards of the Institutional Review Board of Harbin Medical University.

Finally, 296 CRC patients (122 females and 174 males) were included in this study. Patients were followed at 3-6-month intervals in the first year after surgery, and then annually. The last follow-up date was March 15, 2014. At the end of follow-up, 122 (41.22%) patients were confirmed dead, 140 (47.30%) patients were still alive, and 34 (11.48%) patients were lost to follow up (Supplementary Figure 3). The overall survival (OS) time was defined as the length of time from the date of surgery to either the death of patients or the last follow-up time. The disease-free survival (DFS) refers to the period from the date of surgery to the recurrence or metastasis of cancer or the death of patients due to disease progression.

### Genomic DNA extraction and sodium bisulfite modification

Genomic DNA was extracted from tumor tissues and adjacent normal tissues using phenol-chloroform procedure and stored at -80°C [60]. About 2000ng of genomic DNA was subjected to sodium bisulfite modification using the EpiTect Fast DNA Bisulfite kit (Qiagen, Hilden, Germany) according to the manufacturer’s protocol. Human WGA (the whole genome amplified) Methylated and Non-methylated DNA (Zymo Research Corp, Irvine, CA, USA) were converted in the same way as the fully methylated and unmethylated DNA controls. Bisulfited DNA samples were preserved at -20°C for the subsequent experiments avoiding repeated freezing and thawing. The purity and concentration of DNA samples were measured by Nanodrop 2000 spectrophotometer (Thermo Scientific, USA).

### Methylation-sensitive high-resolution melting (MS-HRM)

MS-HRM [61, 62] was performed on LightCycler 480 (Roche Diagnostics, Penzberg, Germany) using matching Multiwell Plate 96. The specific amplified region corresponds to nucleotides 41782664-41782781 (UCSC: hg19 uc004dfl. 4). The amplicon (118bp, including 13 CpG dinucleotides) was located at the promoter of the *CASK* gene. Primers were designed according to the principles outlined in Wojdacz et al. [63] using Primer Premier 5.0 (Premier Biosoft International, Palo Alto, CA). The primer sequences, the mixture for each reaction, and cycling protocol are listed in Supplementary Table 9. The set of methylation standards included 100%, 50%, 25%, 10%, 5%, 1%. Supplementary Figure 4 shows typical MS-HRM melting profiles of the *CASK* promoter region in standard substance. Methylation standards and a non-template control (NTC) were included in each run to control the sensitivity of detection. Additionally, all samples were detected in duplicate. In total, 48 samples (randomly selected from previous batches) were retested at different times to control the reproducibility of detection. We discovered high consistency (κ value = 0.943, *P* < 0.001). Details are shown in (Supplementary Table 10).

The data were analyzed using gene scanning and melting temperature (Tm) calling modules of LightCyler 480 Gene Scanning software version 1.5 (Roche Diagnostics, Penzberg, Germany). The results were jointly determined by two independent operators with the same criteria.

### Definition of methylation status

Heterogeneously methylated amplicons did not melt uniformly and presented with the highly distinct melting curves generated by the heteroduplex formation between strands that only differ at a few CpG sites [40, 45, 55]. MS-HRM can easily distinguish heterogeneous from homogeneous DNA methylation. The methylation profiles were interpreted using both the melting peaks and normalized melting curves [20, 55].

The definition of different methylation status was according to the characteristic melting peaks [64]. Types were as follows: (1) unmethylation (Unm); (2) total positive methylation (Tpm); (3) homogeneous methylation (Hom); (4) total heterogeneous methylation (Thm); (5) early melting heterogeneous methylation 1 (Hem 1-1); (6) early melting heterogeneous methylation 2 (Hem 1-2); (7) cross peak heterogeneous methylation 1 (Hem 2-1); (8) cross peak heterogeneous methylation 2 (Hem 2-2). Table 4 describes the detailed characteristics of different methylation status. The examples of different kinds of methylation profiles detected in our analysis are presented in Figure 1.

**Table 4:** Characteristics of different methylation status.

**Methylation status**	**Characteristics of melting peak**
Unm	Unmethylated peak
Tpm	Thm+Hom
Hom	Homogeneous methylated peak, could be semi-quantitative according to normalized melting curves
Thm	Hem 1-1+Hem 1-2+Hem 2-1+Hem 2-2
Hem 1-1	Methylation melting peak before homogeneous methylation melting profile
Hem 1-2	Methylation melting peak earlier than Hem 1-1
Hem 2-1	Broad melting peak crossing methylation and unmethylation profiles
Hem 2-2	Wider peak than unmethylation peak

### Digital methylation-sensitive high-resolution melting (dMS-HRM) and sequencing

Heterogeneously methylated samples were readily identified using MS-HRM by the characteristic melting patterns. Furthermore, dMS-HRM could provide a visual interpretation of complex methylation patterns [40]. The basis of dMS-HRM is to individually amplify a single DNA template isolated by dilution to discriminate different alleles [65, 66].

Ten-fold serial dilutions of heterogeneously methylated amplicons were made. The replicates of each dilution were amplified to choose the most appropriate dilution for further analysis. Multiple replicates for the selected dilution and methylated control DNA (100% and 0% methylated DNA) were performed using MS-HRM. We analyzed the result to get the digital output of the amplicons. The primer sequence, reaction mixture, and instrument of dMS-HRM were the same with MS-HRM. The cycling protocol of dMS-HRM is shown in Supplementary Table 9.

According to the principle, each well contained no template, single template, or occasionally more than one amplifiable template. And, the expected distribution conformed to Poisson distribution. Amplifications can be easily identified by melting curves with different forms. Melting curves from a single template showed a sharp and smooth single peak. Melting curves generated from multiple templates presented two peaks or more complex patterns.

Replicates with a single sharp melting peak (single template) were selected for sequencing (Sangon Biotech, Shanghai) to visualize the complex heterogeneously methylated amplicons. The sequencing data were analyzed and visualized using the Chromas version 2.6.6 (Technelysium Pty Ltd, South Brisbane, Queensland, Australia).

### Statistical analysis

Means and standard deviations (SD) were calculated for continuous variables. Categorical variables were presented as counts and frequencies, and the different distributions between categorical variables were compared by the Chi-square test. Cox regression model was adopted to estimate the impact of clinicopathologic characteristics and methylation status on patient outcomes. The associations were reported as hazard ratios (HRs) and 95% confidence intervals (CIs). The survival rates were estimated using life table. Survival curves were performed using the Kaplan-Meier method.

The PS-adjusted method, which is a robust method to balance the comparability of multiple confounding variables to achieve an effect similar to randomization in the observational study [39], was adopted to eliminate the potential confounding effects in this study. Moreover, the missing values were complemented by multiple imputations. We further conducted subgroup analysis according to gender, age, tumor site, and Dukes staging. In order to assess the stability of our results, we performed subsequent sensitivity analysis by calculating "confounding RR" which was defined as the ratio of the PS-adjusted HR and the unadjusted HR or the variable-adjusted HR and the unadjusted HR [67].

Besides, we analyzed the relationship between *CASK* methylation status and CRC prognosis in the Cancer Genome Atlas (TCGA) database to validate our results externally. The best cutoff was calculated by X-tile version 3.6.1 (Yale University, USA) [68]. All statistical analyses were performed using SPSS version 23.0 (IBM, Inc, USA). All *P* values were two-sided, and *P* < 0.050 was considered statistically significant.

### Availability of data and materials

All data analyzed in the current study are available from the corresponding author on reasonable request.

## Supplementary Material

Supplementary Figures

Supplementary Tables
