# Supplementary material for: Colorectal cancer patients with CASK promotor heterogeneous and homogeneous methylation display different prognosis
Source: Aging (Albany NY). 2020 Oct 28;12(20):20561–86. doi: 10.18632/aging.103928 (PMC7655177; doi:10.18632/aging.103928)
Supplement: Supplementary Figures [file aging-12-103928-s001..pdf]

SUPPLEMENTARY FIGURES

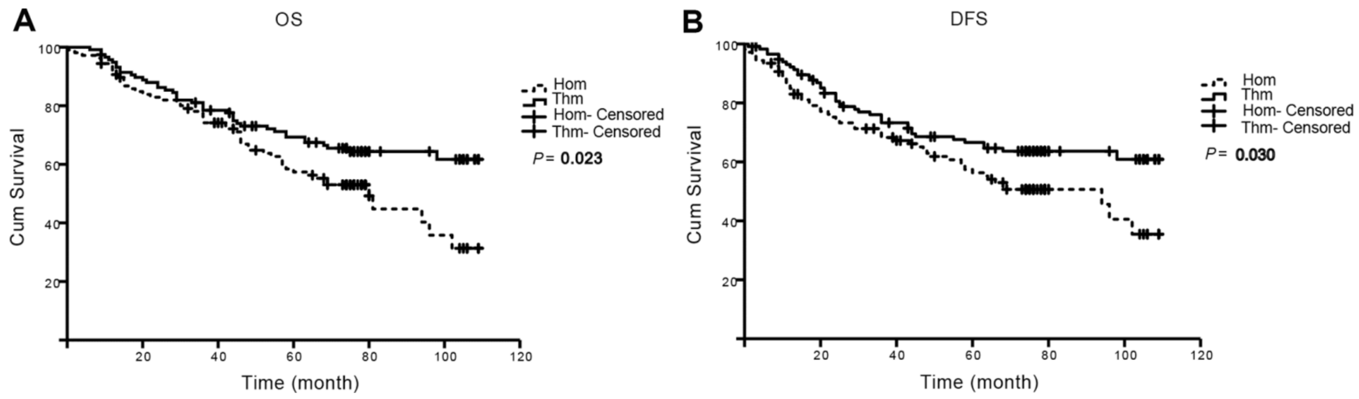

Supplementary Figure 1. Kaplan-Meier curves for survival comparisons of total heterogeneous methylation (Thm) and homogeneous methylation (Hom) group. (**A**) Kaplan-Meier curves for overall survival; (**B**) Kaplan-Meier curves for disease-free survival.

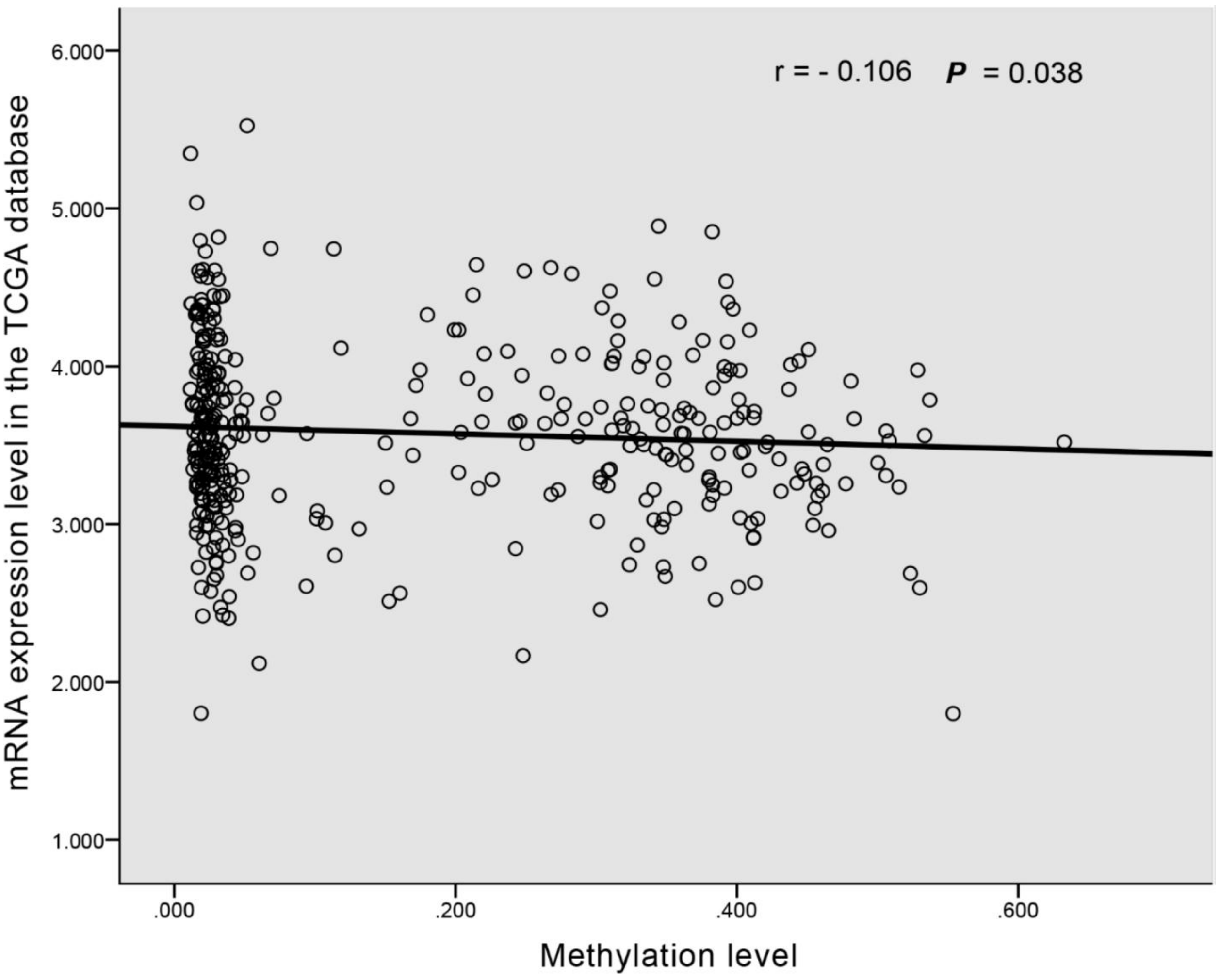

Supplementary Figure 2. Methylation level of *CASK* (cg12614178) and mRNA expression level in the TCGA database.

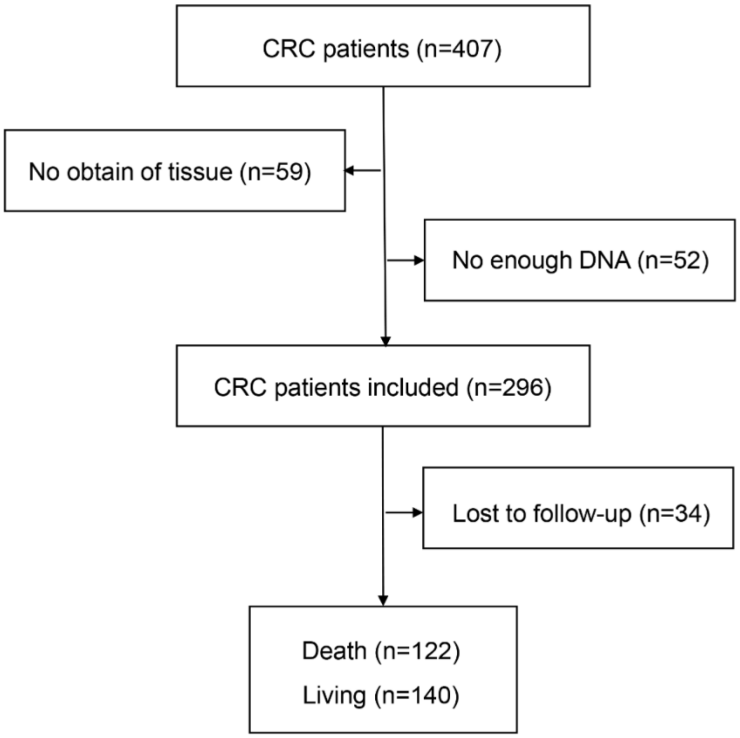

Supplementary Figure 3. Flow chart of participant selection in the cohort study.

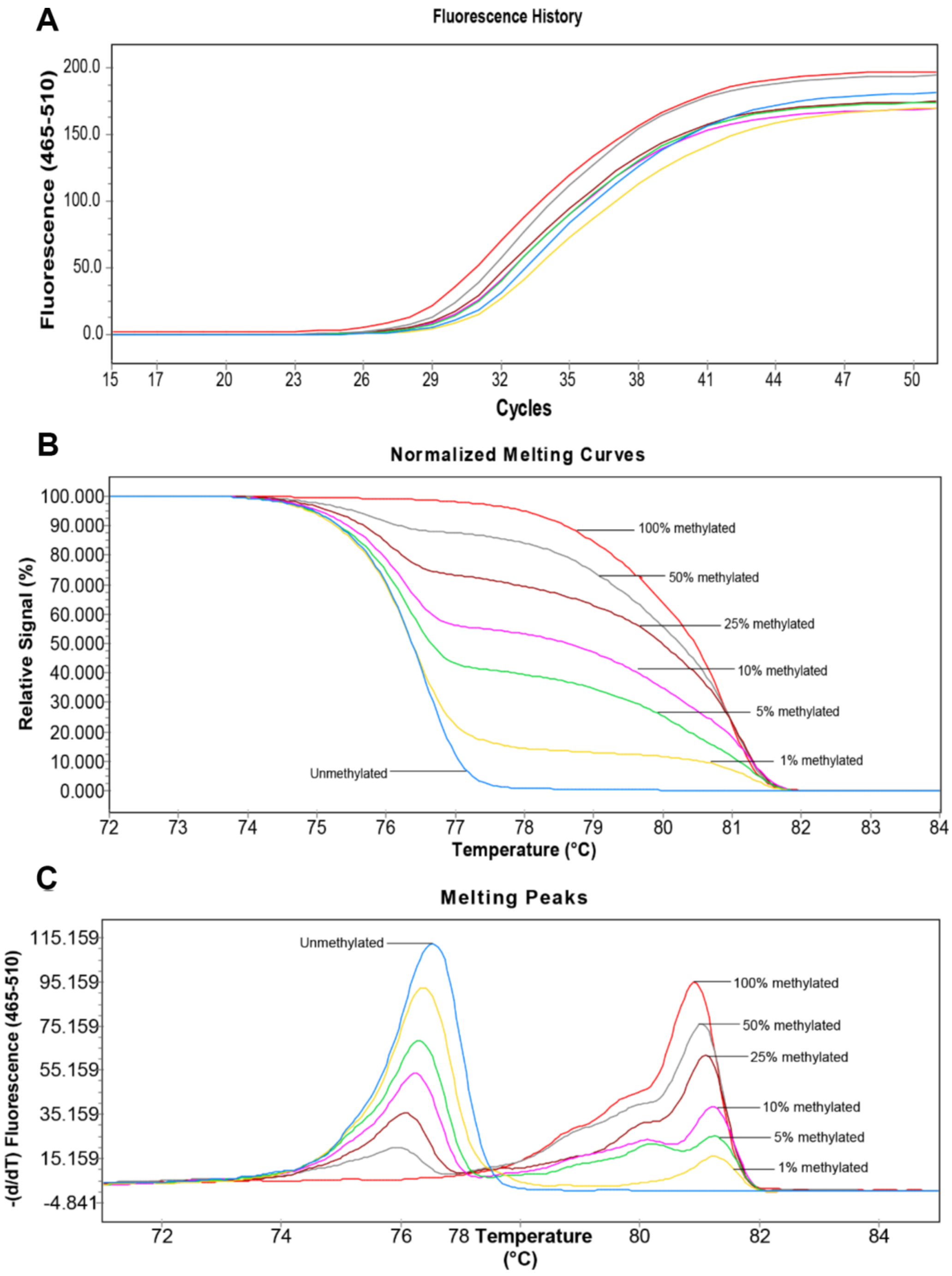

Supplementary Figure 4. High-resolution melting profiles for *CASK* promoter methylation standards. (**A**) Amplification of methylation standard; (**B**) normalized melting curves; (**C**) melting peaks. Standards: 100%, 50%, 25%, 10%, 5%, 1%, 0% methylated DNA.
