# Supplementary material for: Colorectal cancer patients with CASK promotor heterogeneous and homogeneous methylation display different prognosis
Source: Aging (Albany NY). 2020 Oct 28;12(20):20561–86. doi: 10.18632/aging.103928 (PMC7655177; doi:10.18632/aging.103928)
Supplement: Supplementary Tables [file aging-12-103928-s002..pdf]

SUPPLEMENTARY TABLES

Supplementary Table 1. Frequencies of different methylation status in CRC patients.

Methylation status	Tumor tissue (No=296), No (%)	Normal tissue adjacent tumor (No=255), No (%)	$P^{\dagger}$
Unm	72 (24.3%)	82 (32.2%)	
Tpm	224 (75.7%)	173 (67.8%)	**0.041**
Thm	117 (52.2%)	108 (62.4%)	
Hom	107 (47.8%)	65 (37.6%)	**0.042**

†P value was calculated using Pearson's Chi-square test for comparing survival rates between two groups.

Supplementary Table 2. Distribution of the characteristics of CRC patients according to different methylation status (Hom vs Thm).

Baseline characteristics	Total (No=224)	Hom (No=107), No (%)	Thm (No=117), No (%)	$P^{\dagger}$
Age				0.514
< 50	46	20 (18.7%)	26 (22.2%)	
≥ 50	178	87 (81.3%)	91 (77.8%)	
Gender				**< 0.001**
Male	103	20 (18.7%)	83 (70.9%)	
Female	121	87 (81.3%)	34 (29.1%)	
Primary site				0.903
Colon	87	42 (39.3%)	45 (38.5%)	
Rectum	137	65 (60.7%)	72 (61.5%)	
Dukes staging				0.158
A/B	123	64 (59.8%)	59 (50.4%)	
C/D	101	43 (40.2%)	58 (49.6%)	
TNM staging				0.158
I/II	123	64 (59.8%)	59 (50.4%)	
III/IV	101	43 (40.2%)	58 (49.6%)	
Tumor invasion				**0.002**
T1-T3	116	44 (41.1%)	72 (61.5%)	
T4	108	63 (58.9%)	45 (38.5%)	
Lymph node metastasis				0.242
N0	127	65 (60.7%)	62 (53.0%)	
N1/N2	97	42 (39.3%)	55 (47.0%)	
Distant metastasis				0.437
M0	213	103 (96.3%)	110 (94.0%)	
M1	11	4 (3.7%)	7 (6.0%)	
Histological grade				0.641
G1/G2	191	90 (84.1%)	101 (86.3%)	
G3/G4	33	17 (15.9%)	16 (13.7%)	
Histological type				0.142
Adenocarcinoma	168	85 (79.4%)	83 (70.9%)	
Other types	56	22 (20.6%)	34 (29.1%)	
Pathological classification				0.888
Protuberant	156	75 (70.1%)	81 (69.2%)	

Other types	68	32 (29.9%)	36 (30.8%)	
Preoperative CEA				0.263
0-5ng/ml	96	50 (46.7%)	46 (39.3%)	
≥ 5ng/ml	128	57 (53.3%)	71 (60.7%)	
Preoperative CA19-9				0.384
0-37U/ml	159	73 (68.2%)	86 (73.5%)	
≥ 37U/ml	65	34 (31.8%)	31 (26.5%)	

†*P* value was calculated using Pearson's Chi-square test.

Supplementary Table 3. The overall survival rates of CRC patients according to different methylation status.

Methylation status	No (%)	1year		3years		5years		8years	
		SR (96%CI)	*P*†	SR (96%CI)	*P*†	SR (96%CI)	*P*†	SR (96%CI)	*P*†
Unm	72 (24.3%)	0.92 (0.88-0.96)		0.83 (0.73-0.93)		0.71 (0.59-0.83)		0.48 (0.34-0.62)	
Tpm	224 (75.7%)	0.92 (0.88-0.96)	0.620	0.73 (0.67-0.79)	0.482	0.63 (0.51-0.75)	0.372	0.46 (0.38-0.54)	0.881
Hom	107 (47.8%)	0.89 (0.85-0.93)		0.71 (0.61-0.81)		0.57 (0.47-0.67)		0.27 (0.09-0.45)	
Thm	117 (52.2%)	0.94 (0.88-1.00)	0.321	0.76 (0.68-0.84)	0.527	0.69 (0.61-0.77)	0.071	0.56 (0.46-0.66)	**0.023**

†*P* value was calculated using life table.

Supplementary Table 4. Association between *CASK* methylation status and survival of patients with CRC.

Methylation status	No	Multivariate Cox† (OS)		Multivariate Cox† (DFS)	
		HR (95% CI)	*P*‡	HR (95% CI)	*P*‡
Unm	72	1		1	
Tpm	224	0.970 (0.561-1.678)	0.914	0.916 (0.530-1.582)	0.753
Hom	107	2.473 (1.136-5.382)	**0.022**	2.096 (0.969-4.535)	0.060
Thm	117	0.826 (0.451-1.513)	0.536	0.801 (0.439-1.461)	0.469

†Adjusted factors: age, gender, primary site, TNM staging, histological grade, pathological classification, preoperative CA19-9, and postoperative radiotherapy.
‡*P* value was calculated using Cox regression model.

Supplementary Table 5. Associations between *CASK* methylation status, clinicopathologic characteristics and CRC survival in univariate Cox regression models.

Baseline characteristics	Univariate Cox (OS)		Univariate Cox (DFS)	
	HR (95% CI)	*P*[†]	HR (95% CI)	*P*[†]
Age				
< 50	1		1	
≥ 50	0.774 (0.513-1.169)	0.223	0.732 (0.484-1.106)	0.138
Gender				
Male	1		1	
Female	1.140 (0.797-1.632)	0.472	1.138 (0.795-1.628)	0.480
Primary site				
Colon	1		1	
Rectum	1.145 (0.784-1.671)	0.484	1.131 (0.775-1.651)	0.524
Dukes staging				
A/B	1		1	
C/D	2.702 (1.870-3.906)	**< 0.001**	2.628 (1.818-3.799)	**< 0.001**
TNM staging				
I/II	1		1	
III/IV	2.855 (1.967-4.145)	**< 0.001**	2.818 (1.941-4.093)	**< 0.001**
Tumor invasion				
T1-T3	1		1	
T4	1.589 (1.099-2.297)	**0.014**	1.534 (1.062-2.215)	**0.022**
Lymph node metastasis				
N0	1		1	
N1/N2	2.723 (1.882-3.941)	**< 0.001**	2.700 (1.866-3.908)	**< 0.001**
Distant metastasis				
M0	1		1	
M1	5.812 (3.088-10.938)	**< 0.001**	4.550 (2.427-8.533)	**< 0.001**
Histological grade				
G1/G2	1		1	
G3/G4	1.870 (1.191-2.935)	**0.007**	1.933 (1.231-3.036)	**0.004**
Histological type				
Adenocarcinoma	1		1	
Other types	0.876 (0.560-1.369)	0.561	0.851 (0.544-1.330)	0.478
Pathological classification				
Protuberant	1		1	
Other types	2.006 (1.384-2.908)	**< 0.001**	2.017 (1.392-2.933)	**< 0.001**
Preoperative CEA				
0-5ng/ml	1		1	
≥ 5ng/ml	1.973 (1.336-2.913)	**< 0.001**	2.075 (1.405-3.-64)	**< 0.001**
Preoperative CA19-9				
0-37U/ml	1		1	
≥ 37U/ml	5.649 (3.925-8.132)	**< 0.001**	5.815 (4.040-8.371)	**< 0.001**
Postoperative chemotherapy				
No	1		1	
Yes	0.997 (0.698-1.425)	0.988	1.160 (0.811-1.657)	0.416

Postoperative radiotherapy				
No	1		1	
Yes	2.517 (1.308-4.843)	**0.006**	2.860 (1.483-5.518)	**0.002**
Postoperative biotherapy				
No	1		1	
Yes	0.607 (0.347-1.061)	0.080	0.661 (0.378-1.157)	0.147
Methylation status				
Unm	1		1	
Tpm	1.054 (0.695-1.600)	0.803	1.046 (0.689-1.587)	0.833
Thm	1		1	
Hom	1.603 (1.062-2.419)	**0.025**	1.567 (1.039-2.364)	**0.032**

†*P* value was calculated using Cox regression model.

Supplementary Table 6. Association between *CASK* methylation status and survival of patients with CRC.

**Methylation status**	**No**	**Multivariate Cox† (OS)**		**Multivariate Cox† (DFS)**	
		**HR (95% CI)**	***P*‡**	**HR (95% CI)**	***P*‡**
Hom/Thm	107/117	2.501 (1.383-4.525)	**0.002**	2.495 (1.394-4.464)	**0.002**
Hom/Hem1	107/82	2.680 (1.241-5.787)	**0.012**	2.684 (1.252-5.756)	**0.011**
Hom/Hem1-1	107/57	3.473 (1.461-8.253)	**0.005**	3.310 (1.408-3.310)	**0.006**
Hom/Hem1-2	107/25	1.827 (0.663-5.037)	0.244	2.002 (0.726-5.524)	0.180
Hom/Hem2	107/35	3.452 (1.341-8.889)	**0.010**	2.907 (1.161-7.283)	**0.023**
Hom/Hem2-1	107/27	4.836 (1.601-14.608)	**0.005**	3.839 (1.299-11.339)	**0.015**
Hom/Hem2-2	107/8	0.833 (0.175-3.957)	0.818	0.984 (0.276-4.637)	0.984

†Adjusted factors: age, gender, primary site, TNM staging, histological grade, pathological classification, preoperative CA19-9, and postoperative radiotherapy.
‡*P* value was calculated using Cox regression model.

Supplementary Table 7. Association between different methylation status and CRC prognosis in validation dataset with different cutoff value (Hom vs Thm).

**Cutoff value**	**Hom, No**	**Thm, No**	**Univariate Cox (OS)**		**Multivariate Cox† (OS)**	
			**HR (95% CI)**	***P*‡**	**HR (95% CI)**	***P*‡**
Best cutoff	210	169	1.512 (0.976-2.342)	0.064	1.700 (0.819-3.532)	0.155
q25	94	285	1.196 (0.746-1.918)	0.458	0.977 (0.567-1.684)	0.933
q50	189	190	1.348 (0.880-2.065)	0.169	1.219 (0.630-2.358)	0.556
q75	284	95	1.171 (0.715-1.916)	0.532	1.009 (0.519-1.959)	0.980

†Adjusted factors: age, gender, primary site, and histological type.
‡*P* value was calculated using Cox regression model.

Supplementary Table 8. Subgroup analysis of associations between different methylation status and the CRC prognosis in validation dataset (Hom vs Thm).

Subgroup	Hom, No	Thm, No	Univariate Cox (OS)		Multivariate Cox† (OS)	
			HR (95% CI)	P‡	HR (95% CI)	P‡
Gender						
Male	187	19	2.681 (0.651-11.044)	0.172	2.591 (0.624-10.751)	0.190
Female	23	150	1.162 (0.408-3.315)	0.778	1.194 (0.417-3.420)	0.742
Age						
< 50	30	27	0.886 (0.195-4.020)	0.875	0.433 (0.043-4.406)	0.480
≥ 50	180	142	1.513 (0.956-2.395)	0.077	2.038 (0.964-4.306)	0.062
Tumor site						
Rectal	54	40	0.833 (0.313-2.216)	0.715	1.657 (0.343-8.008)	0.530
Colon	156	129	1.777 (1.080-2.921)	**0.024**	1.827 (0.788-4.233)	0.160

†Adjusted factors: age, gender, primary site, and histological type.
‡*P* value was calculated using Cox regression model.

Supplementary Table 9. Primer sequence, reaction mixture, and cycling protocol.

Gene	Direction	Primer sequence	Reaction mixture (10μL)	Cycling protocol
CASK	**Forward**	5′-GGGAGGAGGAGAAAGAGGA-3′	5μL 2×LightCycler 480 High-Resolution Melting Master Mix<br>1.2μL $MgCl_2$ (25 mM)<br>0.25μL of each primer (10 Mm)<br>2.7μL PCR grade water<br>0.6μL bisulfite-modified template DNA (theoretical concentration 25 ng/μL)	**MS-HRM:**<br>**Initial denaturation**: 95°C for 10 min<br>**Cycling**: 55x (95°C for 10s, 57°C for 40s, 72°C for 30s)<br>**Final extension**: 72°C for 10 min<br>**HRM**: 95°C for 1 min, 40°C for 1 min, 69°C-95°C (0.01°C/s)
	**Reverse**	5′-AACCGCGACAAAACCATAAAA-3′		**dMS-HRM:**<br>**Initial denaturation**: 95°C for 10 min<br>**Cycling**: 55x (95°C for 10s, 56°C for 40s, 72°C for 30s)<br>**Final extension**: 72°C for 10 min<br>**HRM**: 95°C for 1 min, 40°C for 1 min, 69°C-95°C (0.01°C/s)

Supplementary Table 10. Results of methylation detection at different time points.

Sample ID	First time	Second time	κ value	$P^{\dagger}$
TN100	Hem 2-1	Hem 2-1	0.943	< **0.001**
TN40	Hem 1-1	Hem 1-1		
23	Hom	Hom		
100	Hom	Hom		
TN23	Hem 2-1	Hem 2-1		
TN25	Hem 1-1	Hem 1-1		
TN94	Unm	Unm		
TN84	Unm	Unm		
TN107	Unm	Unm		
TN86	Hem 1-1	Hem 1-1		
TN108	Hem 2-1	Hem 2-1		
TN112	Hem 1-2	Hem 1-2		
TN151	Unm	Unm		
TN165	Unm	Unm		
TN167	Unm	Unm		
TN126	Hem 2-1	Hem 2-1		
TN120	Hem 2-1	Hem 2-1		
TN127	Hom	Hom		
TN149	Hem 2-1	Hem 2-1		
TN172	Hom	Hom		
TN07365	Unm	Unm		
TN07366	Hom	Hom		
TN07013	Hem 1-2	Hem 1-2		
TN07029	Hom	Hom		
TN189	Hem 2-1	Hem 2-1		
TN07367	Unm	Unm		
TN07031	Hom	Hom		
TN192	Hem 2-1	Hem 2-1		
TN96	Unm	Unm		
TN182	Hem 2-1	Hem 2-1		
TN07354	Unm	Hem 1-1		
TN07004	Hem 1-1	Hem 1-1		
TN07019	Unm	Unm		
TN07355	Hom	Hom		
TN07037	Unm	Unm		
TN07067	Unm	Unm		
TN07038	Unm	Unm		
07104	Hem 1-1	Hem 1-1		
07107	Unm	Hom		
TN07198	Hem 1-2	Hem 1-2		
TN07183	Hem 2-1	Hem 2-1		
TN07221	Hem 2-1	Hem 2-1		
TN07355	Hom	Hom		
TN07382	Hom	Hom		

TN07389	Unm	Unm
TN07381	Unm	Unm
TN07386	Unm	Unm
